# POLYAR, a new computer program for prediction of poly(A) sites in human sequences

**DOI:** 10.1186/1471-2164-11-646

**Published:** 2010-11-19

**Authors:** Malik Nadeem Akhtar, Syed Abbas Bukhari, Zeeshan Fazal, Raheel Qamar, Ilham A Shahmuradov

**Affiliations:** 1 Department of Biosciences, COMSATS Institute of Information Technology, Islamabad, Pakistan; 2 Shifa College of Medicine, Islamabad, Pakistan; 3 Department of Fundamental problems of biological productivity, Institute of Botany, Baku, Azerbaijan

## Abstract

**Background:**

mRNA polyadenylation is an essential step of pre-mRNA processing in eukaryotes. Accurate prediction of the pre-mRNA 3'-end cleavage/polyadenylation sites is important for defining the gene boundaries and understanding gene expression mechanisms.

**Results:**

28761 human mapped poly(A) sites have been classified into three classes containing different known forms of polyadenylation signal (PAS) or none of them (PAS-strong, PAS-weak and PAS-less, respectively) and a new computer program POLYAR for the prediction of poly(A) sites of each class was developed. In comparison with polya_svm (till date the most accurate computer program for prediction of poly(A) sites) while searching for PAS-strong poly(A) sites in human sequences, POLYAR had a significantly higher prediction sensitivity (80.8% versus 65.7%) and specificity (66.4% versus 51.7%) However, when a similar sort of search was conducted for PAS-weak and PAS-less poly(A) sites, both programs had a very low prediction accuracy, which indicates that our knowledge about factors involved in the determination of the poly(A) sites is not sufficient to identify such polyadenylation regions.

**Conclusions:**

We present a new classification of polyadenylation sites into three classes and a novel computer program POLYAR for prediction of poly(A) sites/regions of each of the class. In tests, POLYAR shows high accuracy of prediction of the PAS-strong poly(A) sites, though this program's efficiency in searching for PAS-weak and PAS-less poly(A) sites is not very high but is comparable to other available programs. These findings suggest that additional characteristics of such poly(A) sites remain to be elucidated. POLYAR program with a stand-alone version for downloading is available at http://cub.comsats.edu.pk/polyapredict.htm.

## Background

Polyadenylation including the cleavage of pre-mRNA and adding a stretch of adenosines, poly(A), to the 3'-end is an essential stage of pre-mRNA processing, which results in the generation of the mature mRNA in eukaryotes. This is an important step for the stability, nucleus-to-cytoplasm export and translation initiation of mRNA [1,2]. Polyadenylation is also required for the proper and effective transcription termination, splicing of mRNA, translation termination, as well as being involved in gene silencing [3-8] and genomic imprinting [9]. Although polyadenylation is a common modification of pre-mRNA, it is achieved by different mechanisms in different organisms [10-12]. Moreover, another type of RNA polyadenylation processing with a different set of proteins has been identified recently in eukaryotes, which has been implicated in RNA degradation [8].

mRNA polyadenylation involves (a) a specific endonucleolytic cleavage at the poly(A) site and (b) subsequent addition of a poly(A) tail. In mammals the poly(A) region contains various *cis *acting elements that interact with the corresponding proteins of the mRNA polyadenylation machine, as the cleavage and polyadenylation specificity factor (CPSF), the cleavage stimulation factor (CstF), the cleavage factors I and II (CF I and CF II), and the enzyme poly(A) polymerase (PAP). All components of this machine appear to act cooperatively. In particular, the CPSF and CstF interact with each other and bind to the AAUAAA hexamer (polyadenylation signal, PAS) and its downstream counterpart, U/GU-rich element, respectively [11,13,14].

The PAS is one of the well-studied key elements, which have been shown to be involved in the regulation of mRNA-polyadenylation [15,16]. The optimal (canonical) PAS consists of the AAUAAA motif and most base substitutions in this sequence, except the AUUAAA variant, have been shown to have a significantly reduced cleavage and polyadenylation efficiency (to almost 10% of those sequences with the canonical PAS). However, the AAUAAA element is not as universal a signal as it has previously been considered to be. For example, in human, only ~70% of the 3' expressed sequence tags (ESTs) contain one of the two optimal PAS sequences, AAUAAA or AUUAAA [16,17]. These findings suggest that no consensus sequences of the main cleavage and polyadenylation signals exist, but rather the cooperative action of these sequences and their binding factors results in the pre-mRNA maturation. Indeed, in addition to PAS and the U-/GU-rich elements, a number of auxiliary elements appear to play an important role in the regulation of this process. Comparative studies of the human and *Drosophila melanogaster *polyadenylation regions show that the PAS is highly conserved in these two species. However, as opposed to this, the U-rich downstream sequence element (DSE) shows a higher divergence between the two species. Such a variation of the maturation process of the pre-mRNA elements seems to be related to alternative polyadenylation of the same transcription unit [10,12,14,16,18].

A growing line of evidence indicates that most pre-mRNAs undergo alternative polyadenylation and this mechanism, altogether with alternative transcription initiation and splicing, are used by eukaryotic organisms to produce a diverse number of mature mRNA transcripts from the same transcription unit. This is further confirmed by the fact that splicing and polyadenylation appear to be coupled co-transcriptional events of pre-mRNA maturation. It has been shown that more than half of the human genes have multiple poly(A) sites, which can potentially produce transcripts with variable 3'-untranslated regions (3'-UTRs) potentially encoding in many cases different proteins [7,12,19]. Besides, polyadenylation in the intronic regions can result in conversion of an internal exon to a 3'-terminal exon (termed "composite terminal exon") or usage of a 3'-terminal exon that is otherwise skipped ("skipped terminal exon"; 7,20). It has been shown that about 20% of human genes have, at least, one intron with polyadenylation site(s) which can potentially produce alternative mRNAs encoding different proteins. The conservation of poly(A) signals in introns of mouse and rat genes is lower than that of poly(A) sites in gene ends. Moreover, the intronic polyadenylation activity appears to be dependent on cellular conditions, 5'-splice sites and intron size: larger introns with weaker donor splice sites prevail among exons used as composite terminal exons, whereas skipped terminal exons are associated mostly with strong polyadenylation signals [7,21]. Both bioinformatics and experimental studies indicate that utilizing intronic alternative poly(A) site in human and mouse CstF-77 gene, which encodes one of the three subunits of the CstF, as well as in its *Drosophila *homologue, produces short CstF-77 transcripts lacking sequences encoding some of the functional domains of CstF-77 [20]. Computational analysis of 3'-ends of ESTs suggested the existence of four classes of alternative polyadenylation in human, mouse, and rat: tandem poly(A) sites, composite exons, hidden exons, and truncated exons. It was estimated that about 49% of the human, 31% of the mouse, and 28% of the rat polyadenylated transcription units have alternative polyadenylation sites, which result in the generation of new protein isoforms [19]. Recently, Muro et al. [22] reported that about 60% of human and murine genes utilize multiple (on the average, 3-4) polyadenylation sites. All available data, briefly reviewed above, indicate the importance of accurate identification of polyadenylation sites in genes. Of course, the most accurate approach for the solution of this problem is mapping of the full-length cDNAs onto the genomic sequences. However, till date a complete set of full-length cDNAs is not available, clustering and DNA mapping studies of human and murine EST databases reveals that of the currently known genes, 15% have no EST-supported 3'end. In other words, the 3'-termini of a significant portion of known genes from these species remains to be identified [22]. Similar results have been obtained by Lopez et al. [23]. Therefore, the computational prediction of poly(A) sites becomes very important. However, it is also a challenge for the following reasons: (a) there are gaps in our knowledge on regulation and performance of mRNA polyadenylation; (b) DNA signals and regulatory proteins, involved in the transcription termination, are still poorly described; (c) polyadenylation appears to be tissue- and organism-specific, and also many genes utilize alternative points of polyadenylation; (d) available data of poly(A) sites are very limited: the real transcriptional status of a genome, dependence of cell/tissue/organ, developmental stage and environmental conditions, is unknown; (e) poly(A) sites may exist in coding sequences (CDS) and introns. The last two points create additional problems in getting true positive and negative data sets necessary for learning and testing of any computational tool.

To date, using both experimental and *in silico *mapping of 3'-end ESTs, thousands of poly(A) sites have been identified and analyzed [24,25]. Based on these data, during the last 15 years, a number of attempts have been made to define the consensus sequences involved in the pre-mRNA polyadenylation and to develop computer tools for prediction of poly(A) sites. By analyzing known human poly(A) sites, Yada *et al*. [26] suggested CAAUAAA(U/C) as a consensus of the poly(A) signal. Kondrakhin *et al*. [27] computed a general consensus matrix for 63 poly(A) sites in vertebrate pre-mRNAs and implemented it into the computational method for the recognition of polyadenylation points in mRNA. However, all these methods had a very high false positive rate. Later, Salamov and Solovyev [28], applying a linear discriminant function (LDF)-based algorithm, trained on 131 real poly (A) signal regions and 1466 other regions of human genes with seemingly nonfunctional AAUAAA motif, developed the computer program **POLYAH**. In comparison with the previous analogous methods, the accuracy of this latter approach, which has been tested on a larger data set, was better (sensitivity and specificity of 86% and 51%, respectively). Tabaska and Zhang [29] developed the **polyadq **program, which used two quadratic discriminant functions for the AAUAAA/AUUAAA motifs and a position weight matrix for the downstream elements. In tests on whole genes and the last two exons of genes, polyadq predicted poly(A) signals with a correlation coefficient of 0.413 and 0.512, respectively. Legendre and Gautheret [16] developed the **ERPIN **program based on a probabilistic hidden Markov model, which achieved a prediction specificity of 69 to 85% for a sensitivity of 56%. By analyzing about 4956 EST-validated poly(A) sites from human genes, some sequence determinants of human poly(A) regions were suggested: U-rich upstream and downstream sequence elements (USE and DSE, respectively) appear to be the main characteristics distinguishing true poly(A) sites from randomly occurring A(A/U)UAAA hexamers; while USEs were found to be indiscriminately associated with strong and weak poly(A) sites, DSEs are mostly found near strong poly(A) sites. To recognize poly(A) region of Saccharomyces cerevisiae [30] and Caenorhabditis elegans [31], the weight-matrix-only approaches have been developed. To identify PASs, Bajic et al. [32] have developed another program, **Dragon PolyAtt**, the accuracy of which was substantially better than that obtained by the **polyadq **program. It predicted the two most frequent poly(A) sites, AAUAAA and AUUAAA, with a sensitivity of 48.4% and 13.6%, and specificity of 74% and 79.1%, respectively.

Using a hexamer enrichment method, PROBE, Hu et al. [33] revealed the prevalence of some U-rich and G-rich elements in human poly(A) regions: AUEs (auxiliary USE; -100:-41 region, where +1 is poly(A) site), CUEs (core USE; -40:-1), CDEs (core DSE; +1:+40) and ADEs (auxiliary DSE; +41:+100). Further studies of poly(A) regions by applying position-specific scoring matrixes (PSSM) for these motifs revealed the presence of 15 elements upstream or downstream of the poly(A) site that were suggested to play a role in determining polyadenylation sites.

The latest computational method for the prediction of poly(A) sites is **polya_svm**, which uses support vector machine (SVM) and previously identified 15 cis-motifs for the prediction of the poly(A) sites. Compared with **polyadq**, this method achieves higher sensitivity though with similar specificity [34].

However, there is still room for improvement in the accuracy of these tools, especially for genome-wide searches. In the current work we report the development of a new tool, **POLYAR**, for the recognition of poly(A) sites in human and closely related mammals. The prediction accuracy of this computer program is significantly higher than that of previously developed tools.

## Methods

### Training and testing datasets

Using positions of the human mapped poly(A) sites from polya_DB http://polya.umdnj.edu/PolyA_DB1/ and GenBank annotation of human genome (Build 34.2), 29281 sequences of 600 bp length surrounding the mapped poly(A) site (or Clevage Site, CS) at position 301 (+1) were extracted. To select non-redundant poly(A) sequences, the pairwise comparison of [-50:+50] regions by the BLAST program [35] was performed and the non-redundant positive learning and training dataset of 28761 poly(A) regions showing less than 90% pairwise similarity were selected. These poly(A) regions, here after referred to as "polya DB Set", represent 13693 genes. Applying the same approach, the non-redundant negative set was created. For comparative purposes the polya_svm program [34] was trained and tested on all the 29281 mapped sites.

Studies of PAS motifs upstream of the mapped poly(a) sites revealed 12 different forms of this hexamer; whereas the remaining variations of the PAS-motif have been suggested to be non-functional [17,21]. Out of these 12 forms two PAS-motifs, AAUAAA or AUUAAA, are found in about 70% of the human mapped poly(A) sites. To explain the variations in the PAS-motifs, it was suggested that a site of pre-mRNA cleavage and polyadenylation should be determined by the combination of PAS-motif and some other DNA elements, together with their binding factors [21]. Taking into account these observations and suggestions, and applying Expectation Maximization (EM) approach [36,37], poly(A) sites were differentiated into 3 classes: (1) **PAS-strong **poly(A) sites with the AAUAAA or AUUAAA motifs; (2) **PAS-weak **poly(A) sites with the remaining 10 forms of PAS-motif: AGUAAA, UAUAAA, CAUAAA, GAUAAA, AAUAUA, AAUACA, AAUAGA, ACUAAA, AAGAAA and AAUGAA; (3) **PAS-less **poly(A) sites which lack any of these 12 forms. Such a grouping of poly(A) sites into 3 classes differs from the classification criterion used by Cheng et al. [34]: poly(A) sites are divided into different groups based (a) on their usage (the number of cDNA/ESTs supporting them; "strong", "weak" and "medium" sites) and location ("first", "middle" and "last" upstream sites); as opposed to the classification criterion used in the current study is based only on sequence variation in PAS motifs.

**Figure 1:**
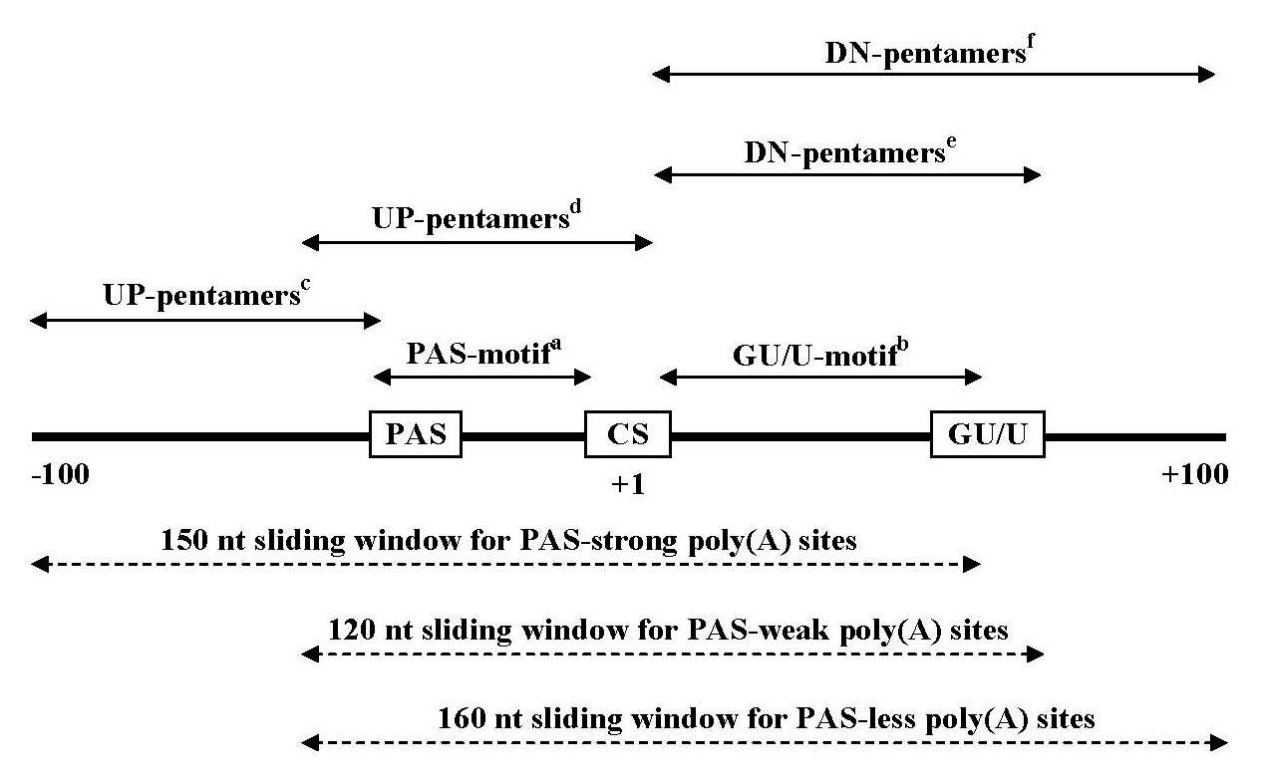
**Schematic presentation of the search regions for PAS-, CS- and GU/U motifs, as well as for upstream (Up)/downstream (DN) pentamers used in the algorithm**. **^a^**[-40:-1], for PAS-strong and PAS-weak poly(A) sites. **^b^**[+2:+50], for all three classes of poly(A) sites. **^c^**60 nt upstream of the PAS-motif's left boundary, for PAS-strong poly(A) sites. **^d^**[-60:-1], for PAS-weak and PAS-less poly(A) sites. **^e^**[+2:+60], for PAS-weak poly(A) sites. **^f^**[+2:+100], for PAS-less poly(A) sites.

Applying our classification criteria, the 28761 sequences were divided into 20225 PAS-strong, 6475 PAS-weak and 2061 PAS-less poly(A) sites. As the training positive datasets, 15000 PAS-strong, 4000 PAS-weak and 1500 PAS-less sequences were taken; the remaining sequences were reserved for the testing procedure. For the training and initial testing, [-100:+50], [-60:+60] and [-60: +100] regions for PAS-strong, PAS-weak and PAS-less poly(A) sites were used, respectively, where different upstream and downstream regions for different PAS groups were selected by criterion based upon Mahalonobis distance and further LDF-scoring for pentamers (Figure 1; Additional files 1 and 2, respectively); for the comparative testing of our approach, the polya_svm http://exon.umdnj.edu/polya_svm and polyadq http://rulai.cshl.org/tools/polyadq/polyadq_form.html programs, these regions were extended up to [-300:+300].

To get a set of negative false poly(A), sequences, the coding sequences (CDS) of only "head-to-head" (H2H) genes annotated (Build 36.3) were extracted, because there is a less chance of the existence of polyadenylation sites in the intergenic spacer between these pairs (in comparison with "tail-to-head" and "tail-to-tail" gene pairs). The middle parts of these CDSs (without the first and last 200 bp) were then divided into non-overlapping fragments of 150, 120 and 160 bp (for PAS-strong, PAS-weak and PAS-less poly(A) sites, respectively) and taken as the negative datasets. Using this method 21,671 sequences ("H2H" negative dataset) were obtained. In addition, using pairwise BLAST comparison non-redundant subset of the H2 H set of 21,350 sequences were obtained. To test the specificity of the current approach, the polya_svm and polyadq programs, we extracted CDSs of 17600 human genes, 19600 human intronic sequences and 3748 5'-UTR regions, and generated 8261 randomized poly (A) site regions (simple randomization by maintaining the same nucleotide frequency as in original poly(A) site sequences).

### Description of the method

To recognize true poly(A) sites, the technique of linear discriminant analysis was used. It was assumed that the site of mRNA cleavage/polyadenylation is determined by some sequence characteristics surrounding the CS, though they may have different weights in the recognition function reflecting their relative significance of recognition. The technique of discriminant analysis allows classification of a given sequence with *p *characteristics (measures) into one of two alternative classes: Class 1 of true poly(A) sites or Class 2 of false poly(A) sites. The procedure of linear discriminant analysis is to find a linear combination (the linear discriminant function, LDF) of these characteristics, that provides maximum discrimination between real and pseudo sites. LDF is given by the formula:

(1) $$Z=\sum_{i=1}^{p}a_{i}x_{i}$$

that classifies given a sequence into Class 1, if z > c, or into Class 2, if *z≤c; x_1_,..., x_k _*are characteristics of used for recognition of a true site. The vector of coefficients $\vec{a}(a_{1},...,a_{p})$ and threshold constant *c *are derived from the training sets by maximizing the ratio of the "between-class" variation of *z *to "within-class" variation and are calculated by the formulas [38]:

(2) $$\vec{a}=s^{-1}({\vec{m}}_{1}-{\vec{m}}_{2})$$

(3) $$c=\vec{a}({\vec{m}}_{1}+{\vec{m}}_{2})/2$$

where ${\vec{m}}_{1}$ and ${\vec{m}}_{2}$ are the sample mean vectors of characteristics for Class 1 and Class 2, respectively; *s *is the pooled covariance matrix of characteristics for Class 1 and Class 2; *s_i _*is covariation matrix, and *n_i _*is the sample size of Class i.

Significance of a given characteristic or set of characteristics can be estimated by the generalized distance between two classes (the Mahalonobis distance, *D^2^*):

(4) $${\vec{D}}^{2}=(\vec{m_{1}-}\vec{m_{2}})s^{-1}(\vec{m_{1}-}\vec{m_{2}})$$

Applying step-wise discriminant procedure for a set of features, suggested to be involved in determination of poly(A) site, for every class of poly(A) sites (PAS-strong, PAS-weak and PAS-less) the subset of significant characteristics significantly increasing the Mahalonobis distance was selected (Table 1).

**Table 1:** Characteristics of polyadenylation regions used for recognition of PAS-strong, PAS-weak and PAS-less sites, and Mahalonobis distance (D2; 38) showing the power of recognition of each characteristic

Characteristics	***D***^***2 ***^**for PAS-strong sites**	***D***^***2 ***^**for PAS-weak sites**	***D***^***2 ***^**for PAS-less sites**
PAS-motif, [-40: -1]	3.18	1.98	
CS-motif*	1.15	1.51	1.36
GU/U-motif, [+1: +50]	1.08	0.90	0.48
Upstream Pentamer Composition	0.72	0.52	0.83
Downstream Pentamer Composition**^c^**		0.84	1.32
PAS-CS distance	1.24	0.77	
CS-GT distance	0.30	0.24	0.17

Total *D^2^*:	7.67	6.75	4.16

* Location of CS-motif: [-15: +3] for PAS-strong and PAS-weak sites; [-9: +3] for PAS-less sites.

#### PAS-motif

Region [-40: -1] was used to search for AAUAAA or AUUAAA forms of PAS-motif with the highest score. An element *w(b, i) *of the signal weight matrix is the frequency of base b *(b=A, U, G or C) *at position i of the PAS-motif calculated on the training samples. Any candidate PAS-motif was scored using the formula:

(5) $$Score(PAS)=\frac{1}{6}\sum_{i=1}^{6}w(b,i)$$

Only sequences with PAS-motif having a score higher than some threshold (in the current case, the minimum score of authentic PAS-motifs) were considered as candidate poly(A) sites.

#### CS-motif

Using position weight matrix of the fixed region, [-15:+3] for the PAS-strong and PAS-weak poly(A) sequences, and [-9:+3] for the PAS-less poly(A) sequences, candidate CS-motifs were scored by the formula:

(6) $$Score(CS)=\frac{1}{k}\sum_{i=1}^{k}w(b,i)$$

where *w(b, i) *is the frequency of base *b *at position *i *of the k-mer candidate CS-motif; *k *= 18 for PAS-strong and PAS-weak sites, and *k *= 12 for PAS-less sites (for CS-motif consensuses see: Additional file 3).

#### GU/U-motif

Position weight matrix for the 10-mer GU/U-motif, obtained from the positive training samples by applying Expectation Maximization [39] in the [+1: +50] region, was used to search for the GU/U-motif with the highest score. Candidate GU/U-motifs were scored by the formula:

(7) $$Score(GU/U)=\frac{1}{10}\sum_{i=1}^{10}w(b,i)$$

where *w(b, i) *is the frequency of base *b *at position *i *of the 10-mer candidate GU/U-motif.

#### Pentamer composition of Upstream and Downstream Regions

Assuming that a priori probabilities for any pentanucleotide in both upstream and downstream regions (in relation to CS) of positive and negative samples are equal, Bayesian probability of observation of pentanucleotide *S_k _(k = 1,1024) *in the corresponding region can be calculated by the following formula:

(8) $$P(S_{k})=\frac{F_{p}(S_{{}_{k}})}{F_{p}(S_{k})+F_{n}(S_{k})}$$

where *F_p_(S_k_) *and *F_n_(S_k_) *are the frequencies of the pentanucleotide S_k _in the corresponding region of positive and negative training samples, respectively. As pentanucleotide preference characteristics of the upstream region, the average value of this probability was taken as follows [40]:

(9) $$Score(UP_{penta})=\frac{1}{56}\sum_{i=1}^{56}P(S_{k,}{}_{i})$$

where *P(S_k, i_) *is *P(S_k_) *for hexamer *S_k _*starting in the position *i *(for pentamers available in 20% or more of the positive set of different classes of poly(A) sequences see Additional files 4, 5 and 6, respectively).

#### Distance between PAS- and CS-motifs

In PAS-strong and PAS-weak poly(A) datasets, authentic PAS-motif shows spatial preferences with respect to the CS. Therefore, the distance between PAS-motif and CS (*D_PAS-Cs_*) is used as one of the significant features for recognition of poly(A) sites. This feature is scored by using frequencies of distances between PAS-motif and CS in the positive training sets:

(10) $$Score(D_{PAS-Cs})=f(D_{PAS-Cs})$$

where *f(D_PAS-Cs_) *is the frequency of a distance betweem PAS-motif and CS observed in sequences from the corresponding positive training dataset.

#### Distance between CS and GU/U-motifs

In all three classes of poly(A) sites, authentic GU/U-motif shows spatial preferences with respect to the CS. Therefore, the distance between CS and GU/U-motif (*D_CS-GU/U_*) was used as one of the valuable features for the recognition of poly(A) sites. This feature is scored by using frequencies of distances between CS and GU/U-motif in the positive training sets:

(11) $$Score(D_{Cs-GU/U})=f(D_{Cs-GU/U})$$

where *f(D_Cs-GU/U_) *is the frequency of a distance betweem CS and GU/U motif observed in sequences from the corresponding positive training dataset.

Most of these features were kept the same for all 3 classes of poly(A) sequences, though there was also some difference, and the discriminating abilities of these features were different (Table 1). In particular, for PAS-strong and PAS-weak poly(A) sequences PAS-motif is the most significant feature.

The total score for every candidate site was computed by formula [1], with the sub-scores for the corresponding features calculated by using the formulas [5-11].

### Estimation of prediction accuracy

Sensitivity (*Sn*), Specificity (*Sp*) and Correlation Coefficient (*CC*) of predictions were calculated by using the following formulas:

(12) $$Sn^{p}=\frac{TP}{TP+FN}$$

(13) $$Sn^{n}=\frac{TN}{TN+FP}$$

(14) $$Sp=\frac{TP}{TP+FP}$$

(15) $$CC=\frac{TP\times TN-FP\times FN}{\sqrt{\left(TP+FP)(TN+FN)(TP+FN)(TN+FP\right)}}$$

where *TP *- True Positives, *TN *- True Negatives, *FP *- False Positives, *FN *- False Negatives, *Sn^p ^*and *Sn^n ^*- Sensitivity of predictions in sets of Positive and Negative samples, respectively.

### Results and Discussion

The algorithm described above was realized in the POLYAR [poly(A) region] program. In the first step, the POLYAR program classifies each position (+1) on a given sequence as a potential CS or non-CS based on three LDF classifiers for PAS-strong, PAS-weak and PAS-less poly(A) sites, with characteristics calculated in the [-100:+50], [-60:+60] and [-60:+100] regions around the current position, respectively (see: Table 1). In the case of PAS-strong and PAS-weak poly(A) sites, only positions with PAS-motif in the region (-40,-1) having a score higher than a preliminary defined threshold are selected for further consideration. First, the LDF for a position is estimated by the classifier for PAS-strong sites; if it is not classified as PAS-strong site, the LDF for that position is estimated by the classifier for PAS-weak sites; otherwise it is estimated by the classifier for PAS-less sites. Estimation of LDFs for the candidate positions is performed by applying thresholds for PAS-strong, PAS-weak and PAS-less poly(A) sites defined on the training dataset. For the final selection of potential CSs, the following criterion is applied:

(i) for any pair of predicted PAS-strong and PAS-weak CSs, or PAS-strong and PAS-weak CSs, within 100 bp of each other, only PAS-strong site is retained;

(ii) for any pair of predicted PAS-weak and PAS-less CSs, within 100 bp of each other, only PAS-weak site is retained;

(iii) for any pair of predicted CSs of the same class, within 100 bp of each other, only the one with the highest score is retained.

The learning and testing procedures were repeated 20 times for all three classes of poly(A) sites. In all these training and testing procedures, randomly created sets of non-CS sequences and the same CS sequences described above were used. For all three classes of poly(A) sites, the same number of sequences from CS and non-CS sets were taken. Results of the initial testing in a single window of [-100:+50], [-60:+60] and [-60:+100] for PAS-strong, PAS-weak and PAS-less poly(A) sites, respectively, are summarized in Table 2.

**Table 2:** Statistics of initial testing of POLYAR program on three classes of CS/poly(A) sites

CS Class	Positive Samples			Negative Samples			CC
	
	TP	FN	Sn,% *	TN	FP	Sn, %*	
PAS-strong (5225 sequences)	4158	1067	79.6	5207	18	99.7	0.81
PAS-week (2475 sequences)	478	1997	19.3	2470	5	99.8	0.32
PAS-less (561 sequences)	93	468	16.5	561	0	100	0.30

* Sensitivity was calculated by formula [12].

Striking differences in the accuracy of predictions in the 3 sets were observed: quite high prediction sensitivity and correlation coefficient for the PAS-strong sequences contradict with very low sensitivity and correlation coefficient for the PAS-weak and PAS-less sequences. Such a variation in accuracy of predictions indicates that we are still far from understanding the regulatory architecture of pre-mRNA 3'-end processing regions lacking the upstream strong PAS site. This gap in our knowledge seems to be a general problem rather than a challenge inherent only in the current approach.

Moreover, the real task of poly(A) site prediction is quite different from just discriminating between CS and non-CS regions: the most probable poly(A) site in a long genomic sequence should be identified. Therefore, we tested the POLYAR recognition algorithm on genomic sequences and compared its performance with polya_svm, till date the most accurate tool for prediction of human poly(A) sites [34], and polyadq program [29]. All three programs were tested on five sets of sequences: (1) [-300:+300] regions relative to the mapped CSs (+1) from 5225 PAS-strong, 2475 PAS-weak and 561 PAS-less test sequences; (2) coding sequences (CDS) of 17600 human genes; (3) 19600 human intronic sequences; (4) 5'-UTR sequences of 3748 human genes; (5) 8261 randomized poly (A) site regions generated by simple randomization of original poly(A) site sequences, with the same nucleotide frequency. Moreover, we tested POLYAR and polya_svm also on [-1000:+1000] gene end (+1) regions of 11916 sequences with the mapped poly(A) site around the [-100:+100] gene end (for comparison of the CPU times used by POLYAR and polya_svm see: Additional file 7).

Table 3 summarizes search results of POLYAR, polya_svm and polyadq on [-300:+300] regions around the mapped PAS-strong, as well as POLYAR and polya_svm on [-300:+300] regions around the mapped PAS-weak and PAS-less poly (A) sites. In these estimations, for POLYAR and polya_svm, a predicted site located within 24 nt (± 12 nt) from a real poly(A) site was considered as a true positive, otherwise it was labeled as a false negative; for polyadq, a predicted site located within 48 nt downstream of a predicted poly(A) signal (PAS) was considered as a true positive, otherwise it was labeled as a false negative.

**Table 3:** Comparative testing results of POLYAR, polya_svm and polyadq rograms in [-300:+300] regions around the mapped PAS-strong, PAS-weak and PAS-less CS/poly (A) sites

Data Set	Programs	TP	FN	FP	Predicted CSs, total	Sn,% *	Sp,% *
PAS-strong (5225 sequences)	POLYAR	4221	1004	2135	6356	80.8	66.4
	polya_svm	3431	1794	3206	6637	65.7	51.7
	polyadq	2804	2421	1177	3981	53.7	70.4
PAS-weak (2475 sequences)	POLYAR	624	1851	1571	2195	25.2	28.4
	polya_svm	645	1830	1417	2185	26.1	29.5
PAS-less (561 sequences)	POLYAR	76	485	544	518	13.5	14.7
	polya_svm	42	519	223	292	7.5	14.4

* Sensitivity and specificity was calculated by formulas [12] and [14], respectively.

For the PAS-strong sites, POLYAR was found to be more sensitive than polya_svm and polyadq (80.8% versus 65.7% and 53.7%, respectively), as well as it being significantly higher in specificity (66.4% versus 51.7%) than polya_svm. At the same time, by comparison with POLYAR, polyadq showed slightly higher specificity (70.4%). The last finding, probably, results from the totally low predictive power of polyadq program. For PAS-weak sites, both POLYAR and polya_svm show very low sensitivity and specificity at almost the same level (25.2% versus 26.1%, 28.4% versus 29.5%, respectively). For PAS-less sites the lowest sensitivity and specificity of prediction by both programs was observed, though the sensitivity of POLYAR was higher (13.5% versus 7.5%).

**Figure 2:**
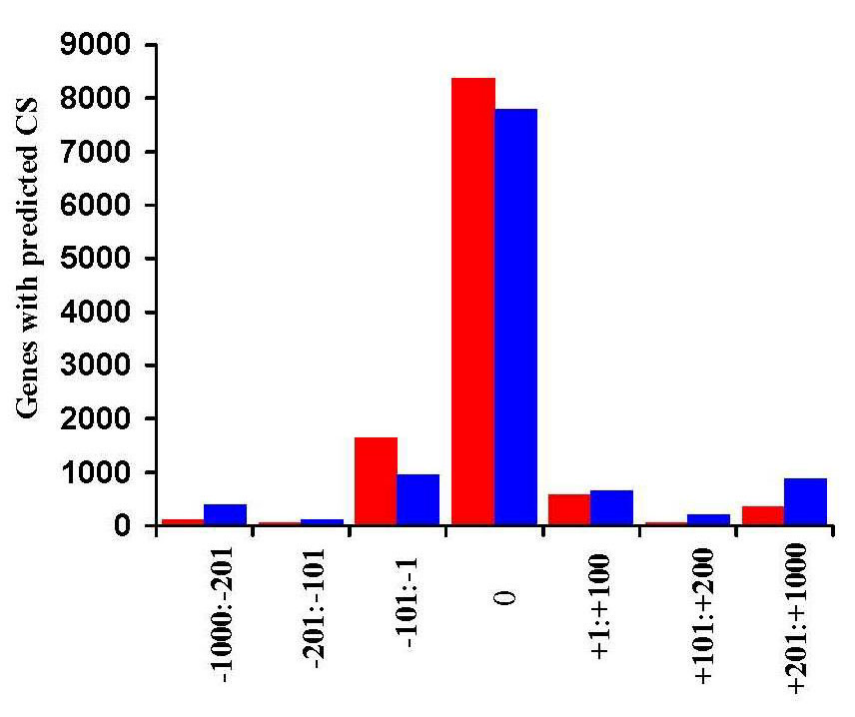
**Location of the nearest CS/poly(A) sites relative to the gene end region (0; ± 12 nt), predicted by POLYAR (red) and polya_svm (blue)**.

Analysis of [-1000:+1000] regions around the annotated end of 11916 genes by POLYAR and polya_svm programs revealed at least one poly(A) site for 11187 (93.9%) and 11013 (92.4%) genes, respectively. With taking only predicted poly(A) sites closest to the gene end, distribution of distances between gene end and predicted cleavage site are presented in Figure 2. Out of the closest CSs, about 95% (10602) and 85% (9407) sites were predicted by POLYAR and polya_svm, respectively, which are located within ± 100 nt around the gene end annotated. POLYAR and polya_svm, altogether, predicted CS within ± 12 nt around the end of 9592 genes; out of these, 6647 sites were predicted by both programs and 1727 and 1148 sites by only POLYAR or polya_svm, respectively. Interestingly, the program shows almost the same accuracy in analysis of mouse and rat gene end sequences (data not shown).

At last, a search for CS/poly(A) sites in the coding sequences of 17600 human genes and 19600 human intronic sequences, as well as in 3748 5'-UTR and 8261 randomized poly (A) site regions was performed (Table 4 and Table 5, as well as Additional files 8 and 9, respectively). In this analysis, under the assumption that CDSs, introns, 5'-UTR and randomized poly(A) regions do not contain real CS(s), false positives and true negatives were computed as number of sequences with at least one site predicted or without any putative site, respectively. In case of CDS sequences, searching for poly(A) sites of all 3 classes altogether, POLYAR and polya_svm programs demonstrated very close sensitivity, though sensitivity of polyadq was higher. However, in a search for PAS-strong or PAS-weak or PAS-less poly(A) sites separately, POLYAR was found to be definitely a more sensitive program, than polya_svm; POLYAR and polyadq showed very close sensitivity. For 5'-UTR sequences, in comparison with polya_svm, sensitivity of POLYAR and polyadq was higher. In case of randomized poly(A) site regions, all 3 programs showed close sensitivity. As to intronic sequences, in comparison with POLYAR, both polya_svm and polyadq show higher sensitivity. In this regard, it should be noted that intronic sequences are, seemingly, not the best candidates for the negative control training and testing. Thus, about 20% of human genes have, at least, one intron with polyadenylation site(s) which can potentially produce alternative mRNAs, though the conservation of poly(A) signals in introns of mouse and rat genes is lower than that of poly(A) sites in gene ends. Moreover, the intronic polyadenylation activity appears to be dependent on cellular conditions, 5'-splice sites and intron size: larger introns with weaker donor splice sites prevail among exons used as composite terminal exons, whereas skipped terminal exons are associated mostly with strong polyadenylation signals [7,21]. Muro et al. [22] found that about 60% of human and murine genes utilize multiple (on the average, 3-4) polyadenylation sites. At last, about 16% of mapped poly (A) sites, used in our studies, are located in introns or internal exons [34].

**Table 4:** Comparative testing results of POLYAR, polya_svm and polyadq programs on coding sequences of 17600 human genes

Programs	TN	FP	**Total**^**5**^	***SN***^**6**^
POLYAR, All**^1^**	13503	4097	7920	76.72%
PAS-strong**^2^**	14091	3509	6005	80.06%
PAS-weak**^3^**	15012	2588	4308	85.30%
PAS_less**^4^**	16699	901	1351	94.88%
polya_svm	13290	4310	6472	75.51%
polyadq	16010	1590	1794	90.97%

**^1 ^**Search for poly(A) sites of all 3 classes. **^2-4 ^**Search for only PAS-strong, PAS-weak and PAS-less poly(A) sites, respectively. **^5 ^**Totally predicted sites. **^6 ^**Sensitivity was calculated by formula [13].

**Table 5:** Comparative testing results of POLYAR, polya_svm and polyadq programs on 19600 intronic sequences

Programs	TN	FP	**Total**^**5**^	***SN***^**6**^
POLYAR, All**^1^**	4581	15019	31998	23.92%
PAS-strong**^2^**	11837	7763	9695	60.39%
PAS-weak**^3^**	9172	10428	14900	46.80%
PAS_less**^4^**	5080	14520	29870	25.92%
polya_svm	13139	6461	8688	67.04%
polyadq	16744	2856	3291	85.43%

**^1-6 ^**See Table 4.

## Conclusions

Searching for PAS-strong CS/poly(A) sites in human sequences, the POLYAR program, as compared to the polya_svm and polyadq programs, had a significantly higher prediction sensitivity (80.8% versus 65.7% and 53.7%, respectively). POLYAR has also higher specificity than polya_svm (66.4% versus 51.7%). However, polyadq showed slightly higher specificity (70.4%) than POLYAR, which may be due to the totally low predictive power of polyadq program. At the same time, in search for PAS-weak and PAS-less CSs both POLYAR and polya_svm programs show very low prediction accuracy. Moreover, POLYAR program shows almost the same accuracy in analysis of mouse and rat gene end sequences (data not shown). These observations indicate that the current knowledge about factors (protein and DNA/RNA sequence requirements) involved in the selection of poly(A) sites is not sufficient to identify polyadenylation regions containing neither AAUAAA nor AUUAAA signals. Therefore, it is important to identify the additional characteristics involved in determining the"PAS-weak" and "PAS-less" CS/poly(A) sites. In particular, we can not exclude that an alternative criterion is required for classification of poly(A) sites with degenerated or without PAS-by signal: there may be different signal(s) which initiate the polyadenylation of pre-mRNA. If this assumption is true, then regrouping of such poly(A) sites into new sub-classes might allow to reveal new conservative motifs around polyadenylation points. Our studies along these lines are continuing.

## Authors' contributions

MNA, SAB and ZF made an equal contribution in collecting dataset of the mapped poly(A) sites and analyzing features significant for recognition of poly(A) sites, as well as in development of the initial, main, body of the POLYAR computer program. IS prepared negative datasets for the training and testing procedures. MNA, SAB, ZF and IS, with an equal contribution, designed a search algorithm. MNA modified and finalized POLYAR program, as well as performed comparative testing of the program. IS and RQ designed and supervised the general strategy of the work and wrote the article. All authors analyzed data, discussed results and approved the final version.

## Supplementary Material

Additional file 1 **Supplemental Table 1 - Mahalonobis distance (D^2^; 38) showing the power of recognition of Upstream Pentamer Composition characteristics in different upstream regions of PAS-strong, PAS-weak and PAS-less sites**. Click here for file

Additional file 2 **Supplemental Table 2 - Mahalonobis distance (D^2^; 38) showing the power of recognition of Downstream Pentamer Composition characteristic in different downstream regions of PAS-weak and PAS-less sites**. Click here for file

Additional file 3 **Supplemental Table 3 - Nucleotide Frequency Matrices for cleavage site (+1) in PAS-strong, PAS-weak and PAS-less poly(A) sequences**. Click here for file

Additional file 4 **Supplemental Table 4 - Upstream pentamers available in 20% or more of the positive set of PAS-strong sequences**. Click here for file

Additional file 5 **Supplemental Table 5 - Pentamers available in 20% or more of the positive set of PAS-weak sequences**. Click here for file

Additional file 6 **Supplemental Table 6 - Pentamers available in 20% or more of the positive set of PAS-less sequences**. Click here for file

Additional file 7 **Supplemental Table 7 - CPU time comparison of POLYAR and polya_svm programs on 8261 poly(A) site and 19600 intronic sequences**. Click here for file

Additional file 8 **Supplemental Table 8 - Comparative testing results of POLYAR, polya_svm and polyadq programs on 3748 5'-UTR sequences**. Click here for file

Additional file 9 **Supplemental Table 9 - Comparative testing results of POLYAR, polya_svm and polyadq programs on 8261 randomized poly(A) site regions**. Click here for file
