# Supplementary material for: POLYAR, a new computer program for prediction of poly(A) sites in human sequences
Source: BMC Genomics. 2010 Nov 19;11:646. doi: 10.1186/1471-2164-11-646 (PMC3053588; doi:10.1186/1471-2164-11-646)
Supplement: Additional file 1 — Supplemental Table 1 - Mahalonobis distance (D2; 38) showing the power of recognition of Upstream Pentamer Composition characteristics in different upstream regions of PAS-strong, PAS-weak and PAS-less sites. [file 1471-2164-11-646-S1.PDF]

Additional file 1:

Supplemental Table 1 - Mahalonobis distance (D^2) showing the power of recognition of Upstream Pentamer Composition characteristics in different upstream regions of PAS-strong, PAS-weak and PAS-less sites

CS Class	D^2 for 60 nt upstream of the PAS-motif's left boundary	D^2 for [-60:-1] upstream of CS	D^2 for [-80:-1] upstream of CS	D^2 for [-100:-1] upstream of CS
PAS-strong	**0.72**	0.70	0.67	0.58
PAS-week	0.48	**0.52**	0.34	0.29
PAS-less		**0.83**	0.81	0.78
