# Supplementary material for: POLYAR, a new computer program for prediction of poly(A) sites in human sequences
Source: BMC Genomics. 2010 Nov 19;11:646. doi: 10.1186/1471-2164-11-646 (PMC3053588; doi:10.1186/1471-2164-11-646)
Supplement: Additional file 2 — Supplemental Table 2 - Mahalonobis distance (D2; 38) showing the power of recognition of Downstream Pentamer Composition characteristic in different downstream regions of PAS-weak and PAS-less sites. [file 1471-2164-11-646-S2.PDF]

Additional file 2:

Supplemental Table 2 - Mahalonobis distance showing the power of recognition of Downstream Pentamer Composition characteristic in different downstream regions of PAS-weak and PAS-less sites

CS Class	D^2 for [+1:+60] downstream of CS	D^2 for [+1:+80] downstream of CS	D^2 for [+1:+100] downstream of CS
PAS-week	**0.84**	0.80	0.74
PAS-less	1.46*	1.37	**1.32**

* When Downstream Pentamer Composition was applied in this region, it results in significant reduction in discrimination ability of GU-rich element (<<0.1) probably due to sharing of similar regions. So, for PAS-less sites we selected [+1:+100] region.
