# Supplementary material for: POLYAR, a new computer program for prediction of poly(A) sites in human sequences
Source: BMC Genomics. 2010 Nov 19;11:646. doi: 10.1186/1471-2164-11-646 (PMC3053588; doi:10.1186/1471-2164-11-646)
Supplement: Additional file 3 — Supplemental Table 3 - Nucleotide Frequency Matrices for cleavage site (+1) in PAS-strong, PAS-weak and PAS-less poly(A) sequences. [file 1471-2164-11-646-S3.PDF]

Additional file 3:

Supplemental Table 3 - Nucleotide Frequency Matrices for cleavage site (+1) in PAS-strong, PAS-weak and PAS-less poly(A) sequences

(a) PAS-strong poly(A) sequences

	-15	-14	-13	-12	-11	-10	-9	-8	-7	-6	-5	-4	-3	-2	-1	+1	+2	+3
A	0.288	0.551	0.479	0.405	0.345	0.309	0.274	0.267	0.289	0.346	0.395	0.330	0.329	0.343	0.413	0.721	0.059	0.336
C	0.195	0.103	0.122	0.137	0.157	0.152	0.148	0.143	0.146	0.148	0.159	0.206	0.193	0.169	0.167	0.097	0.260	0.194
G	0.139	0.111	0.131	0.150	0.157	0.152	0.161	0.155	0.170	0.159	0.138	0.138	0.138	0.182	0.132	0.078	0.246	0.150
T	0.378	0.235	0.269	0.308	0.342	0.386	0.417	0.435	0.395	0.347	0.307	0.326	0.340	0.306	0.287	0.104	0.435	0.320
	**W**	**W**	**W**	**W**	**W**	**W**	**W**	**W**	**W**	**W**	**W**	**n**	**W**	**n**	**W**	**A**	**B**	**W**

(b) PAS-weak poly(A) sequences

	-15	-14	-13	-12	-11	-10	-9	-8	-7	-6	-5	-4	-3	-2	-1	+1	+2	+3
A	0.297	0.460	0.415	0.370	0.324	0.307	0.279	0.287	0.321	0.391	0.440	0.373	0.370	0.408	0.488	0.802	0.036	0.347
C	0.179	0.130	0.138	0.159	0.162	0.155	0.147	0.148	0.153	0.158	0.160	0.197	0.178	0.146	0.147	0.066	0.243	0.177
G	0.156	0.152	0.163	0.158	0.172	0.159	0.169	0.157	0.166	0.156	0.131	0.152	0.142	0.176	0.114	0.064	0.292	0.154
T	0.369	0.258	0.284	0.312	0.342	0.378	0.405	0.408	0.360	0.296	0.270	0.278	0.309	0.271	0.250	0.068	0.428	0.322
	**W**	**W**	**W**	**W**	**W**	**W**	**W**	**W**	**W**	**W**	**W**	**W**	**W**	**W**	**W**	**A**	**B**	**W**

(a) PAS-less poly(A) sequences

	-9	-8	-7	-6	-5	-4	-3	-2	-1	+1	+2	+3
A	0.315	0.227	0.261	0.295	0.337	0.317	0.335	0.315	0.449	0.757	0.043	0.341
C	0.173	0.173	0.171	0.209	0.223	0.235	0.237	0.203	0.175	0.104	0.257	0.193
G	0.151	0.193	0.173	0.177	0.146	0.153	0.148	0.199	0.127	0.07	0.307	0.156
T	0.361	0.407	0.396	0.319	0.293	0.295	0.281	0.283	0.248	0.069	0.393	0.311
	**W**	**w**	**W**	**n**	**n**	**n**	**n**	**n**	**W**	**A**	**B**	**w**
