# Supplementary material for: POLYAR, a new computer program for prediction of poly(A) sites in human sequences
Source: BMC Genomics. 2010 Nov 19;11:646. doi: 10.1186/1471-2164-11-646 (PMC3053588; doi:10.1186/1471-2164-11-646)
Supplement: Additional file 4 — Supplemental Table 4 - Upstream pentamers available in 20% or more of the positive set of PAS-strong sequences. [file 1471-2164-11-646-S4.PDF]

**Additional file 4:**

**Supplemental Table 4 - Upstream pentamers available in 20% or more of the positive set of PAS-strong sequences**

| Pentamer motif | Sequences with at least one occurrence of a pentamer motif (out of 15000) |
|---|---|
| ATTTT | 3987, 26,6% |
| TTTTA | 3680, 24.5% |
| TTTTT | 3551, 23.7% |
| TATTT | 3500, 23.3% |
| TTTGT | 3105, 20.7% |
| TTTAA | 3040, 20.3% |
| TGTTT | 3014, 20.1% |
