# Supplementary material for: POLYAR, a new computer program for prediction of poly(A) sites in human sequences
Source: BMC Genomics. 2010 Nov 19;11:646. doi: 10.1186/1471-2164-11-646 (PMC3053588; doi:10.1186/1471-2164-11-646)
Supplement: Additional file 5 — Supplemental Table 5 - Pentamers available in 20% or more of the positive set of PAS-weak sequences. [file 1471-2164-11-646-S5.PDF]

Additional file 5:

Supplemental Table 5 - Pentamers available in 20% or more of the positive set of PAS-weak sequences

Upstream pentamers		Downstream pentamers	
**Pentamer motif**	**Sequences with at least one occurrence of a pentamer motif (out of 4000)**	**Pentamer motif**	**Sequences with at least one occurrence of a pentamer motif (out of 4000)**
ATAAA	1705, 42.6%	TTTTT	1196, 29.9%
TAAAA	1581, 39.5%	ATTTT	1097, 27.4%
AAAAT	1272, 21.8%	TTTTA	1051, 26.3%
AAATA	1229, 30.7%	TGTTT	1042, 26.1%
AAAAA	1216, 30.4%	TTTCT	1032, 25.8%
TATAA	1133, 28.3%	TATTT	1003, 25.1%
AGAAA	1060, 26,5%	TTTGT	987, 24.7%
ATTTT	1032, 25.8%	TTTTC	951, 23.8%
GTAAA	1017, 25.4	TTTTG	951, 23.8%
TTTTA	1016, 25.4	CTTTT	905, 22.6%
TAAAT	999, 25%	TTATT	884, 22.1%
AATAT	998, 25%	TTTAT	879, 22%
GAAAA	978, 24,5%	GTTTT	876, 21.9%
AAGAA	960, 24%	TCTTT	870, 21.8%
AAATG	957, 23.9%	TTCTT	870, 21.8%
ATATA	951, 23.8%		
TTTTT	946, 23,7%		
TTTAA	934, 23.4%		
TATTT	872, 21.8%		
AAAGA	857, 21.4%		
AGTAA	856, 21.4%		
AAAAG	808, 20.2%		
