# Supplementary material for: POLYAR, a new computer program for prediction of poly(A) sites in human sequences
Source: BMC Genomics. 2010 Nov 19;11:646. doi: 10.1186/1471-2164-11-646 (PMC3053588; doi:10.1186/1471-2164-11-646)
Supplement: Additional file 6 — Supplemental Table 6 - Pentamers available in 20% or more of the positive set of PAS-less sequences. [file 1471-2164-11-646-S6.PDF]

Additional file 6:

Supplemental Table 6 - Pentamers available in 20% or more of the positive set of PAS-less sequences

Upstream pentamers		Downstream pentamers	
**Pentamer motif**	**Sequences with at least one occurrence of a pentamer motif (out of 1500)**	**Pentamer motif**	**Sequences with at least one occurrence of a pentamer motif (out of 1500)**
ATTTT	384, 25.6%	TTTTT	530, 35.3%
TTTAA	379, 25.3%	TTTTG	508, 33.9%
TTTTT	368, 24.5%	TTTGT	494, 32.8%
TTTTA	368, 24.5%	TGTTT	488, 32.5%
TTAAA	360, 24%	TTTCT	486, 32.4%
AAAAA	315, 21%	ATTTT	477, 31.8%
TTTGT	314, 20.9%	TTTTA	460, 30.7%
TGTTT	313, 20.9%	CTTTT	454, 30.3%
TTTCT	311, 20.7%	TATTT	453, 30.2%
TAAAA	309, 20.6%	TCTTT	442, 29.5%
AAAAT	309, 20.6%	TTATT	420, 28%
TTTTC	303, 20.2%	GTTTT	418, 27.9%
		TTTAT	413, 27.5%
		TTCTT	413, 27.5%
		TGTGT	411, 27.4%
		TTTTC	404, 26.9%
		TTGTT	389, 25.9%
		TTTAA	370, 24.7%
		CATTT	345, 23%
		AGAAA	339, 22.6%
		TTCCT	330, 22%
		TTGTG	329, 21.9%
		TTCTG	327, 21.8%
		GAAAA	325, 21.7%
		TCTGT	324, 21.6%
		AAGAA	323, 21.5%
		CTGTG	314, 20.8%
		AAAAT	309, 20.6%
		TGTCT	307, 20.5%
		AAAAG	307, 20.5%
		AAAAA	301, 20.1%
