# Supplementary material for: POLYAR, a new computer program for prediction of poly(A) sites in human sequences
Source: BMC Genomics. 2010 Nov 19;11:646. doi: 10.1186/1471-2164-11-646 (PMC3053588; doi:10.1186/1471-2164-11-646)
Supplement: Additional file 7 — Supplemental Table 7 - CPU time comparison of POLYAR and polya_svm programs on 8261 poly(A) site and 19600 intronic sequences. [file 1471-2164-11-646-S7.PDF]

Additional file 7:

Supplemental Table 7 - CPU time comparison of POLYAR and polya_svm programs on 8261 poly (A) sites and 19600 intronic sequences[1]

Programs	Poly (A) sites	Introns
POLYAR, All[2]	5 min 48.948s	13 min 10.382s
polya_svm	413 min 12.643s	2166 min 49.990s

[1]Technical characteristics of computer used: 2.8 GHZ Intel(R)core(TM)2 Duo CPU E7400 4GB RAM; Operating system: Linux, Fedora 12.

[2] Search for poly(A) sites of all 3 classes.
