# Supplementary material for: POLYAR, a new computer program for prediction of poly(A) sites in human sequences
Source: BMC Genomics. 2010 Nov 19;11:646. doi: 10.1186/1471-2164-11-646 (PMC3053588; doi:10.1186/1471-2164-11-646)
Supplement: Additional file 8 — Supplemental Table 8 - Comparative testing results of POLYAR, polya_svm and polyadq programs on 3748 5'-UTR sequences. [file 1471-2164-11-646-S8.PDF]

Additional file 8:

Supplemental Table 8 - Comparative testing results of POLYAR , polya_svm and polyadq programs on 3748 5'-UTR sequences

Programs	TN	FP	Total[5]	*SN*[6]
POLYAR, All[1]	3214	534	893	85.75 %
PAS-strong[2]	3454	294	372	92.16 %
PAS-weak[3]	3466	282	392	92.48 %
PAS_less[4]	3383	365	645	90.26 %
polya_svm	2960	788	981	78.98 %
polyadq	3605	143	160	96.18%

[1] Search for poly(A) sites of all 3 classes. [2-4] Search for only PAS-strong, PAS-weak and PAS-less poly(A) sites, respectively. [5] Totally predicted sites. [6] Sensitivity was calculated by formula (13).
