# Supplementary material for: POLYAR, a new computer program for prediction of poly(A) sites in human sequences
Source: BMC Genomics. 2010 Nov 19;11:646. doi: 10.1186/1471-2164-11-646 (PMC3053588; doi:10.1186/1471-2164-11-646)
Supplement: Additional file 9 — Supplemental Table 9 - Comparative testing results of POLYAR, polya_svm and polyadq programs on 8261 randomized poly(A) site regions. [file 1471-2164-11-646-S9.PDF]

Additional file 9:

Supplemental Table 9 - Comparative testing results of POLYAR , polya_svm and polyadq programs on 8261 randomized poly (A) site regions (simple randomization by maintaining the same nucleotide frequency as in original poly(A) site sequences)

Programs	TN	FP	Total[5]	*SN*[6]
POLYAR, All[1]	7293	968	1007	88.28 %
PAS-strong[2]	7366	895	929	89.17 %
PAS-weak[3]	7872	389	392	95.29 %
PAS_less[4]	8177	84	85	98.98 %
polya_svm	7958	303	305	96.33 %
polyadq	7993	268	277	96.76%

[1] Search for poly(A) sites of all 3 classes. [2-4] Search for only PAS-strong, PAS-weak and PAS-less poly(A) sites, respectively. [5] Totally predicted sites.

[6] Sensitivity was calculated by formula (13).
